# 2-*O*-*tert*-Butyl­dimethyl­silyl-4,6-*O*-ethyl­idene-*myo*-insitol 1,3,5-orthoformate

**DOI:** 10.1107/S1600536810023214

**Published:** 2010-06-23

**Authors:** Zhouqin Xu, Qiang Wang, Yanchun Sun

**Affiliations:** a The Department of Physics-Chemistry, Henan Polytechnic University, Jiao Zuo 454000, People’s Republic of China; b The Department of Medicine, Hebi College of Vocation and Technology, He Bi 458030, People’s Republic of China

## Abstract

In the title compound, C_15_H_26_O_6_Si, the dioxa six-membered ring bonded to the *myo*-inositol skeleton is in a boat conformation while the rest of the six-membered rings adopt chair conformations.

## Related literature

*myo*-Inositol orthoesters have been used extensively for the synthesis of phospho­inositols and their derivatives, see: Das & Shashidhar (1997 ▶); Sureshan *et al.* (2003 ▶); Potter & Lampe (1995 ▶). For the synthesis of the title compound, see: Li & Vasella (1993 ▶). For a related structure, see: Angyal (2000 ▶). 
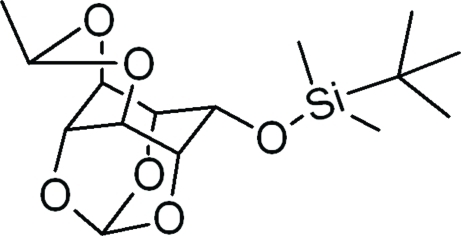


## Experimental

### 

#### Crystal data

C_15_H_26_O_6_Si
*M* *_r_* = 330.45
Orthorhombic, 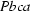

*a* = 12.0170 (4) Å
*b* = 11.2808 (3) Å
*c* = 25.6942 (8) Å
*V* = 3483.14 (18) Å^3^
*Z* = 8
Mo *K*α radiation
μ = 0.16 mm^−1^
*T* = 297 K
0.22 × 0.21 × 0.17 mm

#### Data collection

Bruker APEXII CCD area-detector diffractometer
Absorption correction: multi-scan (*SADABS*; Sheldrick, 2003 ▶) *T*_min_ = 0.966, *T*_max_ = 0.973
55999 measured reflections
4060 independent reflections
2035 reflections with *I* > 2σ(*I*)
*R*_int_ = 0.102

#### Refinement

*R*[*F* ^2^ > 2σ(*F* ^2^)] = 0.052
*wR*(*F* ^2^) = 0.149
*S* = 1.00
4060 reflections
205 parameters
18 restraints
H-atom parameters constrained
Δρ_max_ = 0.24 e Å^−3^
Δρ_min_ = −0.23 e Å^−3^

### 

Data collection: *APEX2* (Bruker, 2007 ▶); cell refinement: *SAINT* (Bruker, 2007 ▶); data reduction: *SAINT*; program(s) used to solve structure: *SHELXS97* (Sheldrick, 2008 ▶); program(s) used to refine structure: *SHELXL97* (Sheldrick, 2008 ▶); molecular graphics: *SHELXTL* (Sheldrick, 2008 ▶); software used to prepare material for publication: *SHELXTL*.

## Supplementary Material

Crystal structure: contains datablocks I, global. DOI: 10.1107/S1600536810023214/pv2294sup1.cif

Structure factors: contains datablocks I. DOI: 10.1107/S1600536810023214/pv2294Isup2.hkl

Additional supplementary materials:  crystallographic information; 3D view; checkCIF report

## Figures and Tables

**Fig. 1.**
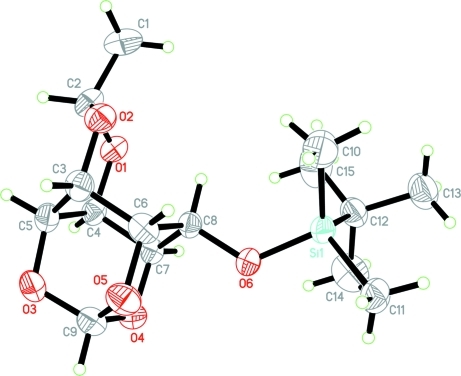
The molecular structure of the title compound, with the atomic-numbering scheme. Displacement ellipsoids are drawn at 30% probability level.

**Fig. 2.**
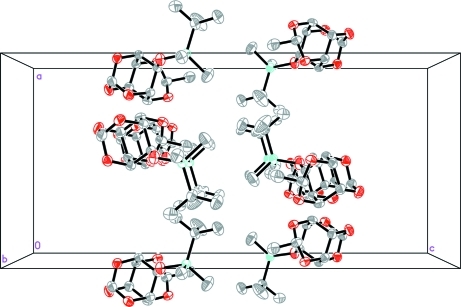
A partial packing diagram of the title compound, viewed down the b-axis.

## supplementary crystallographic information

### Comment

*myo*-inositol orthoesters have been used extensivedly for the synthesis of phosphoinositols (Das & Shashidhar, 1997; Sureshan *et al.*, 2003), their derivatives and other compounds with interesting properties (Potter & Lampe, 1995). We present here the crystal structure of the title compound, which is a key intermediate for the synthesis of phosphorylated *myo*-inositol derivatives (Angyal, 2000).

The bond lengths and angles in the title compound (Fig. 1) are in normal range and agree well with the corresponding bond lengths and angles reported for a related structure (Angyal, 2000). In the title molecule, the six-membered ring containing O1 and O2 is in a boat conformation, the other six-membered rings are in chair conformations. The crystal packing is stabilized by van der Waals forces.

### Experimental

The title compound was prepared according to the literature (Li & Vasella, 1993). Single crystals suitable for X-ray diffraction were prepared by slow evaperation from a solution of ethyl acetate and petroleum ether (1:4).

### Refinement

All H atoms were placed in idealized positions (C—H = 0.98 and 0.96 Å for methyne and methyl H atoms, respectively) and constrained to ride on their parent atoms, with *U*_iso_(H) = 1.2*U*_eq_(methyne C) or 1.5*U*_eq_(methyl C).

### Crystal data

**Table:**

C_15_H_26_O_6_Si	*F*(000) = 1424
*M**_r_* = 330.45	*D*_x_ = 1.260 Mg m^−^^3^
Orthorhombic, *P**b**c**a*	Mo *K*α radiation, λ = 0.71073 Å
Hall symbol: -P 2ac 2ab	Cell parameters from 3612 reflections
*a* = 12.0170 (4) Å	θ = 2.3–18.5°
*b* = 11.2808 (3) Å	µ = 0.16 mm^−^^1^
*c* = 25.6942 (8) Å	*T* = 297 K
*V* = 3483.14 (18) Å^3^	Block, colorless
*Z* = 8	0.22 × 0.21 × 0.17 mm

### Data collection

**Table:**

Bruker APEXII CCD area-detector diffractometer	4060 independent reflections
Radiation source: fine-focus sealed tube	2035 reflections with *I* > 2σ(*I*)
graphite	*R*_int_ = 0.102
φ and ω scans	θ_max_ = 27.6°, θ_min_ = 1.6°
Absorption correction: multi-scan (*SADABS*; Sheldrick, 2003)	*h* = −15→15
*T*_min_ = 0.966, *T*_max_ = 0.973	*k* = −14→14
55999 measured reflections	*l* = −33→32

### Refinement

**Table:**

Refinement on *F*^2^	Primary atom site location: structure-invariant direct methods
Least-squares matrix: full	Secondary atom site location: difference Fourier map
*R*[*F*^2^ > 2σ(*F*^2^)] = 0.052	Hydrogen site location: inferred from neighbouring sites
*wR*(*F*^2^) = 0.149	H-atom parameters constrained
*S* = 1.00	*w* = 1/[σ^2^(*F*_o_^2^) + (0.0588*P*)^2^ + 1.0231*P*] where *P* = (*F*_o_^2^ + 2*F*_c_^2^)/3
4060 reflections	(Δ/σ)_max_ = 0.001
205 parameters	Δρ_max_ = 0.24 e Å^−^^3^
18 restraints	Δρ_min_ = −0.23 e Å^−^^3^

### Special details

**Table:**

Geometry. All e.s.d.'s (except the e.s.d. in the dihedral angle between two l.s. planes) are estimated using the full covariance matrix. The cell e.s.d.'s are taken into account individually in the estimation of e.s.d.'s in distances, angles and torsion angles; correlations between e.s.d.'s in cell parameters are only used when they are defined by crystal symmetry. An approximate (isotropic) treatment of cell e.s.d.'s is used for estimating e.s.d.'s involving l.s. planes.
Refinement. Refinement of *F*^2^ against ALL reflections. The weighted *R*-factor *wR* and goodness of fit *S* are based on *F*^2^, conventional *R*-factors *R* are based on *F*, with *F* set to zero for negative *F*^2^. The threshold expression of *F*^2^ > σ(*F*^2^) is used only for calculating *R*-factors(gt) *etc*. and is not relevant to the choice of reflections for refinement. *R*-factors based on *F*^2^ are statistically about twice as large as those based on *F*, and *R*- factors based on ALL data will be even larger.

### Fractional atomic coordinates and isotropic or equivalent isotropic displacement parameters (Å^2^)

**Table:**

	*x*	*y*	*z*	*U*_iso_*/*U*_eq_	
C1	1.0701 (3)	0.6537 (3)	0.59939 (13)	0.0839 (10)	
H1A	1.1352	0.6900	0.5848	0.126*	
H1B	1.0112	0.7110	0.6013	0.126*	
H1C	1.0473	0.5886	0.5778	0.126*	
C2	1.0960 (2)	0.6094 (2)	0.65278 (12)	0.0623 (8)	
H2	1.1226	0.6746	0.6747	0.075*	
C3	1.2002 (2)	0.4541 (3)	0.69423 (11)	0.0606 (8)	
H3	1.2794	0.4578	0.7032	0.073*	
C4	1.0113 (2)	0.4980 (2)	0.72188 (10)	0.0525 (6)	
H4	0.9641	0.5327	0.7490	0.063*	
C5	1.1318 (2)	0.4988 (3)	0.73907 (11)	0.0615 (7)	
H5	1.1545	0.5797	0.7481	0.074*	
C6	1.1663 (2)	0.3251 (2)	0.68424 (10)	0.0565 (7)	
H6	1.2134	0.2913	0.6569	0.068*	
C7	0.9784 (2)	0.3686 (2)	0.71229 (10)	0.0475 (6)	
H7	0.8989	0.3641	0.7041	0.057*	
C8	1.0450 (2)	0.3186 (2)	0.66778 (10)	0.0477 (6)	
H8	1.0333	0.3668	0.6365	0.057*	
C9	1.1140 (3)	0.3057 (3)	0.77096 (12)	0.0663 (8)	
H9	1.1255	0.2570	0.8021	0.080*	
C10	1.0905 (3)	0.2103 (4)	0.55151 (14)	0.1063 (13)	
H10A	1.0859	0.2952	0.5534	0.160*	
H10B	1.0710	0.1847	0.5171	0.160*	
H10C	1.1651	0.1856	0.5593	0.160*	
C11	1.0206 (3)	−0.0159 (3)	0.60583 (12)	0.0748 (9)	
H11A	1.0988	−0.0286	0.6111	0.112*	
H11B	0.9973	−0.0561	0.5748	0.112*	
H11C	0.9800	−0.0465	0.6351	0.112*	
C12	0.8465 (3)	0.1719 (3)	0.57919 (11)	0.0673 (8)	
C13	0.8201 (3)	0.1084 (3)	0.52793 (12)	0.0919 (11)	
H13A	0.7429	0.1190	0.5196	0.138*	
H13B	0.8359	0.0254	0.5315	0.138*	
H13C	0.8651	0.1411	0.5006	0.138*	
C14	0.7693 (3)	0.1210 (4)	0.62207 (15)	0.1046 (12)	
H14A	0.7834	0.1612	0.6543	0.157*	
H14B	0.7837	0.0378	0.6262	0.157*	
H14C	0.6930	0.1324	0.6122	0.157*	
C15	0.8231 (4)	0.3032 (3)	0.57262 (19)	0.141 (2)	
H15A	0.7474	0.3141	0.5618	0.212*	
H15B	0.8722	0.3354	0.5468	0.212*	
H15C	0.8351	0.3431	0.6052	0.212*	
O1	0.99714 (14)	0.56100 (14)	0.67385 (7)	0.0554 (5)	
O2	1.18006 (14)	0.52151 (17)	0.64779 (7)	0.0621 (5)	
O3	1.14699 (17)	0.42203 (18)	0.78300 (7)	0.0728 (6)	
O4	1.00096 (16)	0.30181 (15)	0.75876 (7)	0.0584 (5)	
O5	1.18069 (15)	0.25908 (18)	0.73137 (8)	0.0689 (6)	
O6	1.01461 (16)	0.19916 (15)	0.65769 (7)	0.0610 (5)	
Si1	0.99324 (6)	0.14381 (6)	0.59918 (3)	0.0530 (2)	

### Atomic displacement parameters (Å^2^)

**Table:**

	*U*^11^	*U*^22^	*U*^33^	*U*^12^	*U*^13^	*U*^23^
C1	0.081 (2)	0.074 (2)	0.096 (3)	0.0003 (17)	0.0099 (19)	0.032 (2)
C2	0.0583 (17)	0.0506 (16)	0.078 (2)	−0.0097 (14)	0.0084 (15)	0.0002 (15)
C3	0.0401 (15)	0.077 (2)	0.0650 (18)	−0.0088 (14)	−0.0059 (13)	0.0029 (16)
C4	0.0549 (16)	0.0477 (14)	0.0550 (17)	−0.0024 (12)	0.0088 (13)	−0.0084 (13)
C5	0.0650 (18)	0.0632 (18)	0.0563 (18)	−0.0168 (15)	−0.0026 (14)	−0.0060 (15)
C6	0.0492 (15)	0.0673 (18)	0.0531 (17)	0.0093 (14)	0.0019 (13)	−0.0007 (14)
C7	0.0458 (15)	0.0451 (14)	0.0515 (16)	−0.0045 (11)	−0.0005 (12)	−0.0021 (12)
C8	0.0507 (15)	0.0432 (14)	0.0491 (16)	−0.0002 (11)	−0.0029 (12)	−0.0048 (12)
C9	0.072 (2)	0.070 (2)	0.0565 (18)	−0.0023 (16)	−0.0084 (16)	0.0069 (16)
C10	0.121 (3)	0.112 (3)	0.086 (3)	−0.040 (2)	0.038 (2)	−0.020 (2)
C11	0.093 (2)	0.0545 (17)	0.077 (2)	0.0151 (16)	−0.0045 (17)	−0.0142 (15)
C12	0.0784 (19)	0.0668 (17)	0.0567 (17)	0.0118 (15)	−0.0106 (15)	−0.0052 (14)
C13	0.104 (2)	0.096 (2)	0.076 (2)	0.0068 (19)	−0.0219 (18)	−0.0122 (18)
C14	0.072 (2)	0.142 (3)	0.100 (2)	0.008 (2)	0.0024 (19)	−0.005 (2)
C15	0.178 (5)	0.084 (3)	0.162 (4)	0.060 (3)	−0.091 (4)	−0.026 (3)
O1	0.0522 (10)	0.0465 (10)	0.0677 (12)	−0.0015 (8)	0.0059 (9)	0.0021 (9)
O2	0.0495 (11)	0.0730 (12)	0.0639 (12)	−0.0066 (9)	0.0089 (9)	0.0042 (11)
O3	0.0812 (14)	0.0844 (15)	0.0527 (12)	−0.0182 (11)	−0.0141 (10)	−0.0024 (11)
O4	0.0626 (12)	0.0582 (11)	0.0544 (12)	−0.0061 (9)	0.0037 (9)	0.0049 (9)
O5	0.0637 (12)	0.0759 (13)	0.0671 (14)	0.0167 (10)	−0.0079 (10)	0.0074 (11)
O6	0.0870 (14)	0.0419 (10)	0.0543 (11)	−0.0050 (9)	−0.0042 (10)	−0.0059 (8)
Si1	0.0636 (5)	0.0452 (4)	0.0502 (5)	−0.0004 (4)	0.0056 (4)	−0.0067 (3)

### Geometric parameters (Å, °)

**Table:**

C1—C2	1.493 (4)	C9—O5	1.397 (3)
C1—H1A	0.9600	C9—O3	1.406 (3)
C1—H1B	0.9600	C9—H9	0.9800
C1—H1C	0.9600	C10—Si1	1.852 (3)
C2—O1	1.415 (3)	C10—H10A	0.9600
C2—O2	1.421 (3)	C10—H10B	0.9600
C2—H2	0.9800	C10—H10C	0.9600
C3—O2	1.436 (3)	C11—Si1	1.840 (3)
C3—C5	1.502 (4)	C11—H11A	0.9600
C3—C6	1.533 (4)	C11—H11B	0.9600
C3—H3	0.9800	C11—H11C	0.9600
C4—O1	1.434 (3)	C12—C15	1.516 (4)
C4—C5	1.513 (4)	C12—C13	1.532 (4)
C4—C7	1.533 (3)	C12—C14	1.550 (5)
C4—H4	0.9800	C12—Si1	1.864 (3)
C5—O3	1.434 (3)	C13—H13A	0.9600
C5—H5	0.9800	C13—H13B	0.9600
C6—O5	1.432 (3)	C13—H13C	0.9600
C6—C8	1.520 (3)	C14—H14A	0.9600
C6—H6	0.9800	C14—H14B	0.9600
C7—O4	1.437 (3)	C14—H14C	0.9600
C7—C8	1.505 (3)	C15—H15A	0.9600
C7—H7	0.9800	C15—H15B	0.9600
C8—O6	1.420 (3)	C15—H15C	0.9600
C8—H8	0.9800	O6—Si1	1.6480 (18)
C9—O4	1.395 (3)		
			
C2—C1—H1A	109.5	O4—C9—H9	107.7
C2—C1—H1B	109.5	O5—C9—H9	107.7
H1A—C1—H1B	109.5	O3—C9—H9	107.7
C2—C1—H1C	109.5	Si1—C10—H10A	109.5
H1A—C1—H1C	109.5	Si1—C10—H10B	109.5
H1B—C1—H1C	109.5	H10A—C10—H10B	109.5
O1—C2—O2	111.3 (2)	Si1—C10—H10C	109.5
O1—C2—C1	107.8 (2)	H10A—C10—H10C	109.5
O2—C2—C1	107.4 (2)	H10B—C10—H10C	109.5
O1—C2—H2	110.1	Si1—C11—H11A	109.5
O2—C2—H2	110.1	Si1—C11—H11B	109.5
C1—C2—H2	110.1	H11A—C11—H11B	109.5
O2—C3—C5	111.6 (2)	Si1—C11—H11C	109.5
O2—C3—C6	108.6 (2)	H11A—C11—H11C	109.5
C5—C3—C6	107.5 (2)	H11B—C11—H11C	109.5
O2—C3—H3	109.7	C15—C12—C13	108.8 (3)
C5—C3—H3	109.7	C15—C12—C14	109.3 (3)
C6—C3—H3	109.7	C13—C12—C14	108.3 (3)
O1—C4—C5	111.2 (2)	C15—C12—Si1	111.8 (3)
O1—C4—C7	107.6 (2)	C13—C12—Si1	110.7 (2)
C5—C4—C7	107.4 (2)	C14—C12—Si1	107.9 (2)
O1—C4—H4	110.2	C12—C13—H13A	109.5
C5—C4—H4	110.2	C12—C13—H13B	109.5
C7—C4—H4	110.2	H13A—C13—H13B	109.5
O3—C5—C3	109.3 (2)	C12—C13—H13C	109.5
O3—C5—C4	110.4 (2)	H13A—C13—H13C	109.5
C3—C5—C4	107.3 (2)	H13B—C13—H13C	109.5
O3—C5—H5	109.9	C12—C14—H14A	109.5
C3—C5—H5	109.9	C12—C14—H14B	109.5
C4—C5—H5	109.9	H14A—C14—H14B	109.5
O5—C6—C8	109.0 (2)	C12—C14—H14C	109.5
O5—C6—C3	108.7 (2)	H14A—C14—H14C	109.5
C8—C6—C3	110.3 (2)	H14B—C14—H14C	109.5
O5—C6—H6	109.6	C12—C15—H15A	109.5
C8—C6—H6	109.6	C12—C15—H15B	109.5
C3—C6—H6	109.6	H15A—C15—H15B	109.5
O4—C7—C8	109.6 (2)	C12—C15—H15C	109.5
O4—C7—C4	108.5 (2)	H15A—C15—H15C	109.5
C8—C7—C4	109.9 (2)	H15B—C15—H15C	109.5
O4—C7—H7	109.6	C2—O1—C4	114.9 (2)
C8—C7—H7	109.6	C2—O2—C3	114.5 (2)
C4—C7—H7	109.6	C9—O3—C5	110.8 (2)
O6—C8—C7	110.9 (2)	C9—O4—C7	110.8 (2)
O6—C8—C6	110.0 (2)	C9—O5—C6	110.5 (2)
C7—C8—C6	106.3 (2)	C8—O6—Si1	124.48 (16)
O6—C8—H8	109.8	O6—Si1—C11	104.99 (12)
C7—C8—H8	109.8	O6—Si1—C10	110.57 (14)
C6—C8—H8	109.8	C11—Si1—C10	110.20 (17)
O4—C9—O5	112.5 (2)	O6—Si1—C12	109.54 (12)
O4—C9—O3	110.7 (2)	C11—Si1—C12	111.17 (15)
O5—C9—O3	110.5 (2)	C10—Si1—C12	110.25 (17)
			
O2—C3—C5—O3	174.7 (2)	C1—C2—O2—C3	−169.0 (2)
C6—C3—C5—O3	55.7 (3)	C5—C3—O2—C2	−4.0 (3)
O2—C3—C5—C4	54.9 (3)	C6—C3—O2—C2	114.4 (2)
C6—C3—C5—C4	−64.1 (3)	O4—C9—O3—C5	−62.2 (3)
O1—C4—C5—O3	−172.03 (19)	O5—C9—O3—C5	63.1 (3)
C7—C4—C5—O3	−54.5 (3)	C3—C5—O3—C9	−59.7 (3)
O1—C4—C5—C3	−52.9 (3)	C4—C5—O3—C9	58.1 (3)
C7—C4—C5—C3	64.6 (3)	O5—C9—O4—C7	−59.7 (3)
O2—C3—C6—O5	−176.93 (19)	O3—C9—O4—C7	64.4 (3)
C5—C3—C6—O5	−56.1 (3)	C8—C7—O4—C9	59.0 (3)
O2—C3—C6—C8	−57.5 (3)	C4—C7—O4—C9	−61.0 (3)
C5—C3—C6—C8	63.4 (3)	O4—C9—O5—C6	60.5 (3)
O1—C4—C7—O4	175.20 (18)	O3—C9—O5—C6	−63.7 (3)
C5—C4—C7—O4	55.3 (3)	C8—C6—O5—C9	−59.9 (3)
O1—C4—C7—C8	55.4 (3)	C3—C6—O5—C9	60.3 (3)
C5—C4—C7—C8	−64.5 (3)	C7—C8—O6—Si1	135.56 (19)
O4—C7—C8—O6	61.8 (3)	C6—C8—O6—Si1	−107.1 (2)
C4—C7—C8—O6	−179.1 (2)	C8—O6—Si1—C11	154.8 (2)
O4—C7—C8—C6	−57.9 (3)	C8—O6—Si1—C10	35.9 (2)
C4—C7—C8—C6	61.2 (3)	C8—O6—Si1—C12	−85.8 (2)
O5—C6—C8—O6	−61.8 (3)	C15—C12—Si1—O6	65.4 (3)
C3—C6—C8—O6	179.0 (2)	C13—C12—Si1—O6	−173.1 (2)
O5—C6—C8—C7	58.4 (3)	C14—C12—Si1—O6	−54.8 (2)
C3—C6—C8—C7	−60.8 (3)	C15—C12—Si1—C11	−179.0 (3)
O2—C2—O1—C4	53.3 (3)	C13—C12—Si1—C11	−57.5 (3)
C1—C2—O1—C4	170.8 (2)	C14—C12—Si1—C11	60.8 (3)
C5—C4—O1—C2	0.2 (3)	C15—C12—Si1—C10	−56.5 (3)
C7—C4—O1—C2	−117.2 (2)	C13—C12—Si1—C10	65.0 (3)
O1—C2—O2—C3	−51.2 (3)	C14—C12—Si1—C10	−176.7 (2)
